# Research on Bending Creep Test and Long-Term Creep Behavior Prediction of Asphalt Concrete

**DOI:** 10.3390/ma18102381

**Published:** 2025-05-20

**Authors:** Yue Zhu, Changhong Yang, Zimo Zhong, Changsheng Huang, Yingbo Zhang, Shan Feng, Shutian Li, Rengui Jiang

**Affiliations:** 1 School of Civil Engineering and Architecture, Xi’an University of Technology, Xi’an 710048, China; ych085900@163.com (C.Y.); 17852022156@163.com (C.H.); zhangyb68@163.com (Y.Z.); list@xaut.edu.cn (S.L.); jrengui@163.com (R.J.); 2 College of Water Conservancy and Civil Engineering, South China Agricultural University, Guangzhou 510642, China; 13926160795@163.com; 3 Hydraulic Asphalt Techniques Institute, Xi’an University of Technology, Xi’an 710048, China; fengshan@xaut.edu.cn

**Keywords:** asphalt concrete facing, bending creep test, Findley model, long-term creep behavior

## Abstract

Different temperatures and continuous loads have significant effects on the long-term performance of asphalt concrete facings. The effects of temperature and stress on creep strain and creep rate were analyzed by designing a bending creep test of impermeable asphalt concrete under different temperatures and stresses. Based on the test data, a time–temperature–stress-dependent creep constitutive model was constructed to predict the long-term creep behavior of asphalt concrete at low temperature. The results showed that the creep behavior of asphalt concrete showed significant temperature and stress dependence. The creep behavior accelerated as the temperature or stress increased, especially under high-stress conditions, indicating obvious nonlinear characteristics. Under the condition of 0.2376 MPa, when the temperature increased from 0 °C to 20 °C, the strain at the creep time of 9330 s nearly increased by 24 times. Under 0 °C, the loading stress increased from 0.2376 MPa to 1.3176 MPa, and the strain nearly increased by six times at a creep time of 880 s. The creep strain is expected to increase to 8% after 8 years at −15 °C and 0.2376 MPa. The results can provide a scientific basis for engineering practice and significant implications for designing and maintaining asphalt concrete facings.

## 1. Introduction

Under the background of carbon peaking and carbon neutrality, pumped storage power stations have developed rapidly to achieve energy saving and emission reduction. As the pumped storage power station in the operation process needs to experience the cycle of the cyclic water storage and release process, the structure must withstand repeated deformation, which puts forward strict requirements on the ability of the material to adapt to the deformation. Asphalt concrete, owing to its superior impermeability, durability, and deformation adaptability, has emerged as the preferred material for impermeable structures in hydraulic engineering. At present, more and more projects are adopting asphalt concrete facings as impermeable bodies. As the key impervious structure of hydraulic construction, the stability and durability of asphalt concrete facings are crucial to ensure the safe operation of the whole structure. With climate change and the emergence of severe weather, the influence of temperature on asphalt concrete facings is becoming increasingly significant. Creep behavior, representing the typical mechanical response of asphalt concrete under coupled sustained loading and temperature effects, fundamentally embodies the viscoelastic nature of this material. Asphalt concrete facings are continuously subjected to complex environmental actions such as hydrostatic pressure, mechanical loads, and thermal cycling. The gradual accumulation of deformations under these conditions may lead to structural failure and seepage issues. Recently, extensive research has been conducted by scholars on the creep behavior of asphalt concrete under temperature–load coupling effects, establishing a comprehensive theoretical framework to elucidate its mechanistic evolution [1,2,3].

Asphalt concrete belongs to temperature–viscoelastic materials, and the time–temperature superposition principle (TTSP) is an important theory for studying the mechanical behavior of viscoelastic materials across temperature scales [4]. Zhang et al. conducted compression and tensile tests on asphalt concrete specimens in different ranges of the temperature strain rate, and the results showed that the TTSP applied to the modulus and strength values [5]. Wang et al. investigated the stress–strain–strength behavior of asphalt concrete at different temperatures and shear strain rates by applying the time–temperature superposition principle to establish the domain of shear modulus and shear strength values of asphalt concrete, and comparing the shear modulus and shear strength values of asphalt concrete under different loading conditions [6]. Teltayev et al. studied the steady-state creep rate of asphalt concrete in detail within the range of stress changes and temperature changes, and described the dependence of steady-state creep rate on stress with a power function at all test temperatures [7]. Iskakbayev et al. studied the steady-state creep process of asphalt concrete at different temperatures and different stresses, and the results showed that stress has a great influence on the creep characteristics of asphalt concrete, and a change of one order of magnitude in stress will lead to a change in magnitude of creep rate [8]. The application of the time–temperature superposition principle relies on the linear viscoelastic assumption and does not integrate the influence of the nonlinear viscoelastic change of asphalt concrete on the time–temperature equivalence relationship. There is significant uncertainty in the long-term performance prediction of the creep behavior of asphalt concrete, and its prediction accuracy has certain limitations. Although the above-mentioned research conducted a systematic study on the creep behavior of asphalt concrete through the TTSP and the power function model, it still focused on using a single principle or model for the research, making it difficult to reflect the complex engineering environment faced by hydraulic asphalt concrete facings.

In light of the inherent limitations of the time–temperature superposition principle in characterizing the nonlinear viscoelastic behavior of asphalt concrete, domestic and international researchers have recently developed diverse constitutive models through multiscale experimental methodologies and advanced material constitutive theories. These models, constructed from multidimensional perspectives, aim to comprehensively elucidate the creep deformation mechanisms of asphalt concrete under conditions of fluctuating temperatures and applied stress loading. Yu et al. conducted bending creep tests at different temperatures and stresses, used the Burgers model to describe the viscoelastic behavior of hydraulic asphalt concrete, nonlinearly fitted the data by the fastest descending iterative method, obtained the fitted curves and model parameters, and analyzed the pattern of change in parameters [9]. Nguyen proposed an improved nonlinear viscoelastic model and investigated the nonlinear behavior of asphalt mixtures under small strain. The results found that this model has excellent performance in describing the nonlinear behavior of asphalt mixtures under small strain [10]. Costanzi et al. developed a constitutive model for an asphalt matrix, which included strain rate and the temperature effect [11]. Bai et al. adopted a series of short-term creep tests under various temperatures and stress levels, combined with the time–temperature superposition principle [12]. To study the linear viscoelastic behavior of a glass–asphalt mixture, Arabani et al. investigated the combined effects of applied stress and temperature on creep behavior, subsequently proposing the first constitutive model for the viscoelastic response of glass–asphalt mixtures. This model was formulated to characterize creep deformation mechanisms under variable loading frequencies and thermal conditions [13]. Using creep testing methodologies, Al-Qadi calibrated parameters for a time-hardening model and implemented these parameters within a finite element framework. The results demonstrated that the nonlinear time-hardening creep model could effectively predict critical rutting damage in hot-mix asphalt (HMA) induced by diverse tire configurations, as well as shear creep strains localized at the edges of tire contact patches [14]. Asphalt concrete is a time–temperature–stress-dependent viscoelastic material, and its mechanical properties and durability will change significantly under different temperatures and continuous loads. While prior research has analyzed the creep deformation behavior of asphalt concrete through distinct constitutive modeling frameworks, these studies predominantly focused on isolated thermal or nonlinear mechanical regimes. Specifically, investigations were confined to either temperature-dependent constitutive models or stress-dominated nonlinear formulations, with limited integration of interactive effects. Consequently, conventional modeling paradigms governed by singular influencing factors fail to authentically replicate the material’s performance under complex thermo-mechanical coupling conditions. This methodological fragmentation results in theoretical frameworks that exhibit an insufficient correlation with practical engineering requirements, particularly in scenarios characterized by concurrent thermal gradients and mechanical loading histories.

At present, the temperature effect has not been combined with the nonlinear behavior of asphalt concrete in creep behavior research, there is a lack of multi-field coupling conditions of asphalt concrete creep models, and more focus is needed on the short-term creep behavior of asphalt concrete at different temperatures and stress levels under the prediction of long-term creep performance, making it difficult to better reveal the creep damage of asphalt concrete facings in their lifespan. It is difficult to better reveal the change rule of asphalt concrete facings during their lifespan, and this cannot accurately provide theoretical reference for engineering practice. Therefore, in order to more accurately and effectively simulate and predict the actual mechanical response of asphalt concrete, and to reveal the change rule of its long-term performance under different temperatures and loads, this paper takes the asphalt concrete facings of pumped storage power stations as the research object, and combines the temperature effect with the nonlinear behavior to simulate the complex environment faced by the asphalt concrete facings in engineering practice. Breaking through the limitation of the traditional model that only considers a single factor, a time–temperature–stress-dependent creep constitutive model is constructed, the bending creep test is carried out to clarify the viscoelastic temperature effect, and the nonlinear response mechanism of asphalt concrete is systematically characterized under different temperatures and stresses. Finally, based on model simulations, long-term creep behavior under low-temperature/high-stress conditions is predicted, with the objective of revealing the creep damage evolution patterns of asphalt concrete facings throughout their operational lifespan.

## 2. Theoretical Background

### 2.1. The Time–Temperature–Stress Superposition Principle

**Figure 1:**
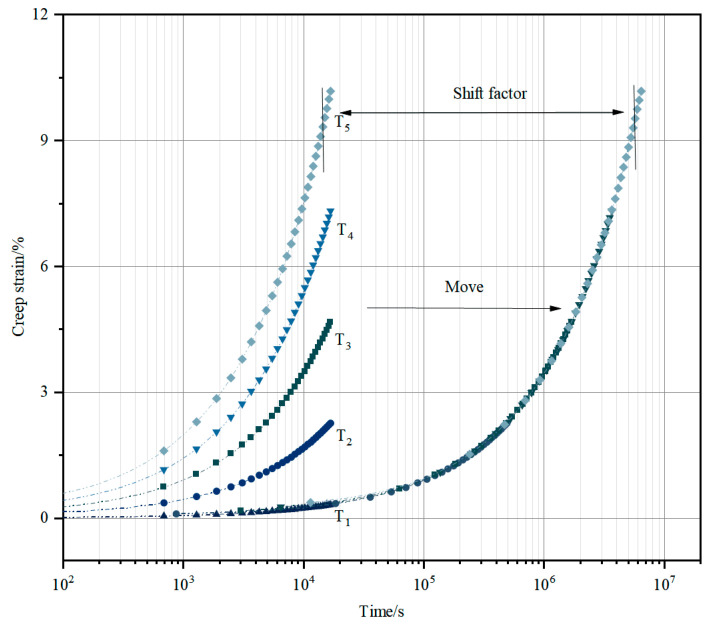
Time–temperature equivalence principle.

The TTSP is shown in Figure 1. The creep experiment is carried out at different temperatures to predict the long-term creep behavior of asphalt concrete at low temperatures [15]. Firstly, a reference temperature is obtained as a benchmark based on the validation of frost crack test data carried out in a number of actual projects, and the short-term creep curves at other temperatures are moved horizontally on the logarithmic time axis using the temperature displacement factor, thus overlapping into the corresponding main creep curve at the reference temperature. The creep behavior of asphalt concrete at the target reference temperature can be preliminarily predicted using the main curve at the reference temperature [16]. Based on the actual needs of the project, 0 °C is determined as the target reference temperature. Calculation and analysis show that the calculated stress value under this target reference temperature setting is more conservative than the actual working conditions, which can provide the necessary safety reserves for the engineering structure and meet the strict requirements for the reliability of mechanical properties in the design of hydraulic structures.

The creep strain ε(T,t) at increasing temperatures is equivalent to the creep curve obtained at the reference temperature T_1_ moving log*φ_T_* in the direction of increasing logarithmic time, which is defined as follows [17]:

(1) $$\varepsilon (T,\;t)=\;\varepsilon (T_{1},\frac{t}{\phi_{T}})\;=\varepsilon (T_{1},\;\log t+\log\phi_{T})$$

In the formula, the following are defined:

*ε*(*T*, *t*)—creep strain, %;

*T*_1_—reference temperature, °C;

*t*—time, s;

*φ_T_*—temperature shift factor.

The temperature displacement factor *φ_T_* is a function of temperature. For polymer materials, the William–Landel–Ferry (WLF) equation is used to calculate the displacement factor [18]. It is defined as follows:

(2) $$\log\phi_{T}=\frac{-C_{1}(T\;-\;{\;T}_{0})}{C_{2}+(T\;-{\;T}_{0})}$$

In the formula, the following are defined:

C_1_ and C_2_—material constants, where the unit of C_2_ is °C;

*T*_0_—reference temperature, °C;

*T*—experiment temperature, °C.

The Time–Stress Superposition Principle (TSSP) is also used to predict long-term creep behavior [19]. The method uses short-term creep data at higher stress levels to predict long-term creep behavior at lower stress levels at constant temperatures. With the TSSP, researchers can accelerate the creep experimenting process while maintaining accurate predictions of the material’s long-term properties. The expression of creep strain is defined as follows:

(3) $$\varepsilon (\sigma ,\;t)=\varepsilon (\sigma_{0},\;\frac{t}{\phi_{\sigma }})=\varepsilon (\sigma_{0},\;\log t+\log\phi_{\sigma })$$

By moving the short-time creep curve along the logarithmic time axis at a constant temperature, the main creep curve and the corresponding displacement factor are obtained. It is defined as follows [20]:

(4) $$\log\phi_{\sigma }=\frac{{-C}_{1}(\sigma \;-\;\sigma_{0})}{C_{3}+(\sigma \;-\;\sigma_{0})}$$

In the formula, the following are defined:

C_1_ and C_3_—material constants, where the unit of C_3_ is °C;

*σ*_0_—reference stress, MPa;

*σ*—experimental stress, MPa;

*φ_σ_*—stress displacement factor.

In the prediction of the long-term creep behavior of viscoelastic materials, the Time–Temperature–Stress Superposition Principle (TTSSP) provides a method to quantify the relationship between time, temperature, and stress. This principle states that the effect of a change in temperature or stress on the creep response is equivalent to a shift on a logarithmic time scale [14]. Using this principle, the short-term creep curves obtained under different temperature or stress conditions can be equivalent to the principal curves of the reference temperature and stress by horizontal displacement. The time–temperature–stress displacement factor log*φ_Tσ_* is defined as follows:

(5) $$\log\phi_{T\sigma }=\log\phi_{T}+\log\phi_{\sigma }$$

### 2.2. Creep Constitutive Model

The creep constitutive model integrates a variety of creep theories and is applied to specific materials to deepen the understanding and prediction of material creep behavior. It can more accurately predict the risk of creep deformation and instability of asphalt concrete, guide the design and use of materials, and thus ensure the safety of engineering construction and subsequent operations. As a classical creep constitutive model, the Bailey–Norton model is characterized by its simple structure and takes stress and time as independent parameters [21]. It is defined as follows:

(6) $$\varepsilon (t)\;={\;A}_{1}\sigma^{m}t^{n}$$

In the formula, the following are defined:

A_1_—coefficients related to material properties and temperature, MPa^−m^·h^−n^ (the time unit is h) or MPa^−m^·s^−n^ (the time unit is s);

*m*—positive real number, called the stress index;

*n*—a positive real number, called the time index;

*σ*—stress applied in the span of the specimen, MPa.

The creep conforming to Formula (6) is called power law creep. Under low-temperature or high-stress conditions, the power law relation fails, and the relation between creep and stress is modified as follows:

(7) $$\varepsilon (t)={\;A}_{2}\exp^{\left(B\sigma \right)}t^{n}$$

A_2_ and B are stress-independent coefficients. Equations (6) and (7) can be summarized as follows:

(8) $$\varepsilon (t)=A_{3}{(\sin h(\alpha \sigma ))}^{m}t^{n}$$

A_3_ and α are stress-independent coefficients, but temperature-dependent.

The Arrhenius formula is added to the constitutive model to obtain the time–temperature–stress-dependent creep constitutive model. The Arrhenius formula is shown in Equation (9).

(9) $$k\;=A\exp(-\frac{E_{a}}{RT})$$

In the formula, the following are defined:

*k*—reaction rate constant, with units dependent on the reaction type (e.g., s⁻^1^, mol/(L·s)).

*A*—pre-exponential factor; its units match those of *k*.

*E_a_*—activation energy, J/mol.

R—universal gas constant, J/(mol·K).

*T*—absolute temperature, K.

This model is generalized from the Findley model and constitutes a general Findley constitutive model, which comprehensively describes the creep behavior of materials under different temperature and stress conditions [22]. It is defined as follows:

(10) $$\varepsilon (t)=A\exp(-\frac{Q_{c}}{RT}){(\sin h(\alpha \sigma ))}^{m}t^{n}$$

In the formula, the following are defined:

*A*—coefficients related to material properties and stresses;

R—universal gas constant, J/(mol·K);

*T*—absolute temperature, K;

*Q_c_*—apparent activation energy of creep, J/mol;

## 3. Material and Methods

### 3.1. Test Materials and Equipment

#### 3.1.1. Test Materials

The coarse and fine aggregates used in this experiment were all from a material factory near a pumped storage power station in Zhangye City, Gansu Province of China. The mineral aggregate was limestone particles sized between 0.075 and 19 mm, and the filler was ground limestone. The asphalt adopts Karamay 70# A-Grade petroleum asphalt, and the quality tests of raw materials were conducted using the Test code for hydraulic asphalt concrete (DL/T 5362-2018). The results show that the quality of the raw materials meets the corresponding specifications [23]. The basic parameters of coarse aggregate, fine aggregate, filler, and asphalt are shown in Table 1, Table 2, Table 3 and Table 4.

Among them, due to the superior performance of K70 asphalt, no fracture occurred after the ductility reached 100 cm.

**Figure 2:**
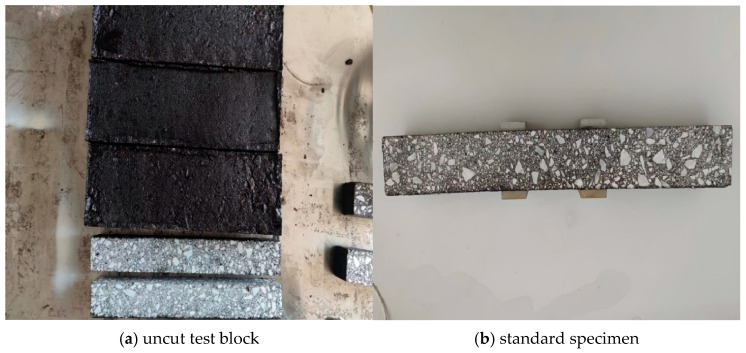
Asphalt concrete material.

The test materials used in the test are shown in Figure 2, where Figure 2a is the uncut test block, and Figure 2b is the standard specimen after the test block is cut into 500 mm × 50 mm × 50 mm and maintained at constant temperature.

#### 3.1.2. Test Equipment

**Figure 3:**
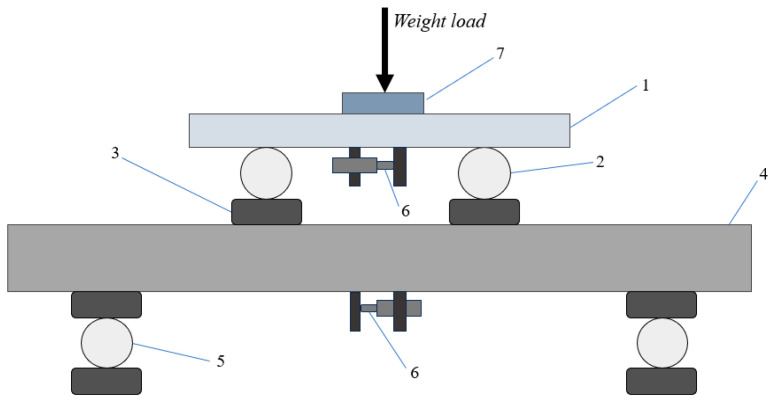
Schematic diagram of four-point bending test. 1—spread beam; 2—bending roller; 3—rubber pad; 4—asphalt concrete specimen; 5—support roll; 6—displacement sensor; and 7—dead weight.

The test equipment used a self-designed four-point bending creep experimenter; the model of the equipment is LMT-2, which can withstand a load range of 0~100 kN. The specimen is placed on the lower ends of the support rollers, and the center distance between the two support rollers is nearly 30 cm. Regarding the upper ends of the bending rollers, the center distance between the two bending rollers is about 14 cm. To prevent damage to the specimen during the test under the action of a load and bending rollers, bending rollers and support rollers in contact with the specimen position were padded with rubber pads. Test stress was applied through top loading weight control and load transfer through the spread beam, connected to thermostatically controlled water bath equipment with a temperature control accuracy of ±0.5 °C. The displacement measurement was performed using Solartron Metrology’s OP/6.0 high-accuracy displacement sensor, model MO0060JCL03AV10-02, with an acquisition accuracy of 0.001 mm. The four-point bending experiment schematic is shown in Figure 3.

### 3.2. Design of Bending Creep Test

To study the creep characteristics of the impervious layer of the asphalt concrete facing under the action of temperature and stress, the asphalt concrete test block was cut into a sample of 500 mm × 50 mm × 50 mm, and the sample was placed in the bending creep tester at a constant temperature for 12 h, and then loaded after the temperature was stabilized. The test temperatures are −20 °C, −15 °C, −10 °C, −5 °C, 0 °C, 5 °C, 10 °C, 15 °C, and 20 °C to fully cover the typical low-temperature-to-normal-temperature range. Because the bending creep test results are greatly affected by stress level, selecting too low a stress level will lead to slow creep development, which brings errors in deflection measurement and makes data analysis difficult. Due to excessive stress, deformation develops rapidly, and creep is not easily observed. Generally, under the action of appropriate external stresses, a more obvious creep phenomenon can be observed. According to the four-point bending calculation principle, the loading stress calculation formula is defined as follows:

(11) $$\sigma =F(\frac{3(L_{s}-{\;L}_{b})}{2Bh^{2}})+\frac{3\rho gL_{s}^{2}}{4h}$$

In the formula, the following are defined:

*σ*—load stress, MPa;

*F*—mid-span load (weight load and the dead weight), N;

*L_s_*—the distance between the center lines of two support rollers, mm;

*L_b_*—the distance between the center lines of two bending rollers, mm;

*B*—sample width, mm;

*h*—sample thickness, mm;

*ρ*—specimen density, kg·m^−3^.

According to the Test code for hydraulic asphalt concrete (DL/T 5362-2018), the creep load, divided into 3 to 5 grades, can be tested at 20% to 80% of the maximum strength of the specimen. The specimen is subjected to the set constant load, which remains unchanged, and is loaded until the specimen is damaged or its deformation is stabilized [23]. The stress–strain characteristics of asphalt concrete are related to temperature, and stress softening will occur at 0 °C [24]. Therefore, considering the measurement accuracy and observation requirements, 7 stress levels were selected for loading under the condition of 0 °C in this experiment to deeply analyze the influence of loading stress on bending creep. For other temperature conditions, three stress levels were selected for loading according to the bending strength at the corresponding temperature to study the influence of temperature on bending creep [25]. Due to the influence of the dead weight of the four-point bending creep tester, the lowest loading stress is 0.2376 MPa, and the remaining stresses are progressively loaded by adjusting the weight of the weights. Table 5 details the flexural creep experiment loads at each temperature.

## 4. Results and Discussion

### 4.1. Analysis of Bending Creep Test of Asphalt Concrete

#### 4.1.1. Creep Response Analysis

**Figure 4:**
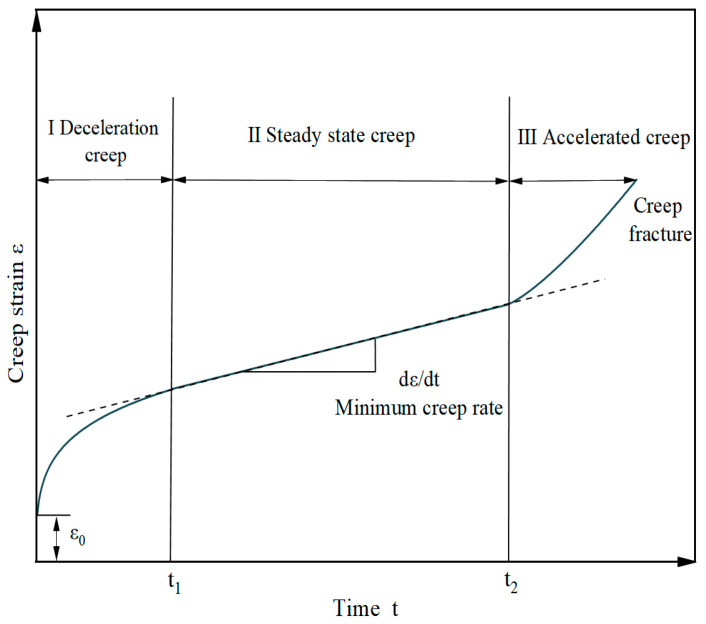
Typical creep curve.

Like most viscoelastic materials, the creep process of asphalt concrete can be divided into three stages: The deceleration creep stage is mainly elastic strain and recoverable viscoelastic strain. In the steady creep phase, the creep rate is constant and the deformation is irreversible. During the accelerated creep phase, the molecular chain breaks and the creep rate increases rapidly, eventually leading to material destruction [13]. This characteristic creep curve of viscoelastic materials is shown in Figure 4.

**Figure 5:**
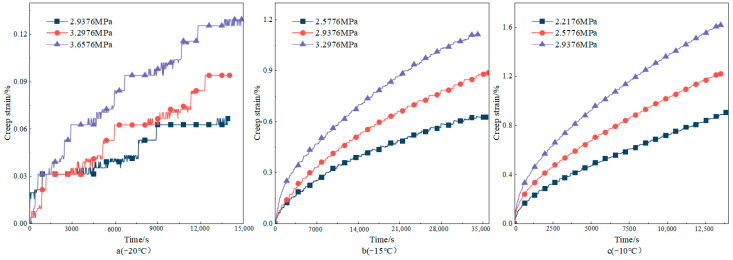

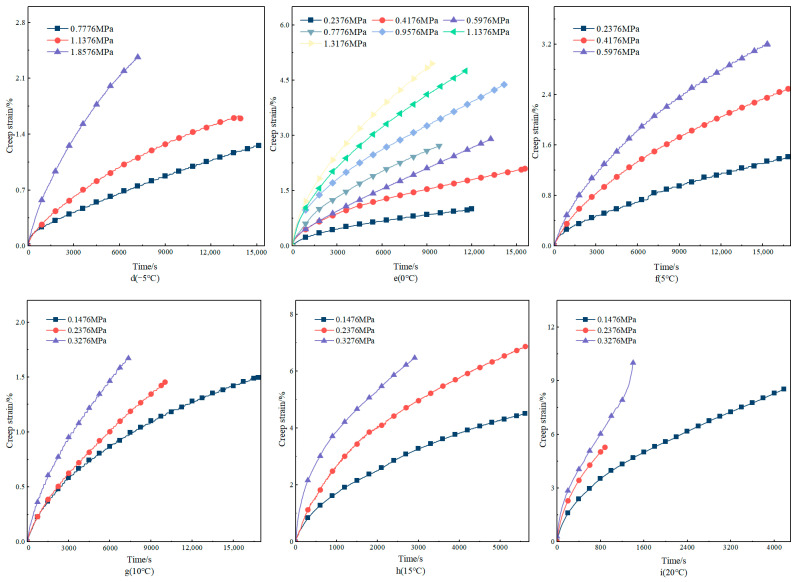
Different stress–creep curves under different temperature conditions.

In the bending creep test under the condition of −20–20 °C, the creep curve obtained by the test is shown in Figure 5. The test results show that, except for the sample loaded at 0.3276 MPa at 20 °C, which entered the accelerated creep stage, the rest of the tested samples were still in the steady creep region at the end of the test. This phenomenon indicates that at relatively high temperatures and specific stresses, the internal structural changes of asphalt concrete are intensified, creep development is accelerated, and the deformation rate increases rapidly with time. Under the condition of −20 °C, the creep curve shows obvious volatility due to the slow progress of creep. It is mainly affected by the superposition of the characteristics of asphalt concrete itself and the test measurement error. Asphalt concrete is a viscoelastic material; at low temperatures, the viscosity of asphalt increases, the adhesion with the mineral powder is enhanced, and the relative displacement between the aggregates is difficult, resulting in an extremely slow creep deformation process of asphalt concrete, and it is difficult for the test instrument to accurately record its small deformation changes. At the same time, systematic errors accumulated and superimposed during the long time and low-rate creep measurement process, which interfered with the accuracy of the data, and thus led to the obvious curve fluctuation.

The level of applied stress has a significant effect on the creep strain of asphalt concrete under equal creep time and temperature conditions. Taking the creep test at 0 °C as an example, the creep curve increases with the increase in stress. The test end time (9330 s) at the loading of 1.3176 MPa is taken as the observation point. The creep strains of specimens under 0.2376 MPa, 0.4176 MPa, 0.5976 MPa, 0.7776 MPa, 0.9576 MPa, 1.1376 MPa, and 1.3176 MPa are 0.870%, 1.571%, 2.169%, 2.644%, 3.331%, 4.182%, and 4.952%. This result shows that the creep strain increases with the increase in stress level. When the loading stress increases from 0.2376 MPa to 1.3176 MPa, the stress increases by about six times, and the corresponding creep strain nearly increases by six times, which is an approximately equal proportion increase. This indicates that in a certain stress range, the creep strain of asphalt concrete and the stress level show an approximately linear relationship; the increase in stress directly leads to the corresponding increase in creep strain, so stress is the key factor driving the creep deformation of asphalt concrete. The nonlinear phenomenon is more obvious at high stress levels. For example, under the condition of a high-stress-level creep test at −15 °C, creep strain increases with the increase in stress and experiences an accelerated increase. This phenomenon indicates that when the stress exceeds a certain threshold, the intermolecular interactions within the asphalt concrete are further destroyed, and its viscoelastic properties undergo significant changes, with a relative increase in the viscous component, which leads to a more rapid increase in the deformation rate with the increase in stress, presenting a clear pattern of nonlinear change in the law. The nonlinear phenomenon is more obvious at high stress levels. For example, in the creep test of a high stress level at −15 °C, the creep strain increases rapidly with the increase in stress. Creep compliance is the ratio of creep strain to stress. Calculated when the loading stress is 2.5776 MPa, 2.9376 MPa, and 3.2976 MPa, the creep compliance at loading 30,000 s is 0.2312 MPa^−1^, 0.2750 MPa^−1^, and 0.3205 MPa^−1^, respectively. The creep compliance increases with the increase in stress. Therefore, the creep strain of asphalt concrete is nonlinear [26]. In summary, the relationship between creep strain and the stress level of asphalt concrete presents different patterns of change under different stress levels. Under the low stress level, the creep behavior of asphalt concrete approximates a linear change relationship. Meanwhile, under the high stress level, the viscoelastic properties of asphalt concrete are changed, and the creep softness also changes with the stress, showing an obvious nonlinear change relationship.

#### 4.1.2. Creep Rate Analysis

**Figure 6:**
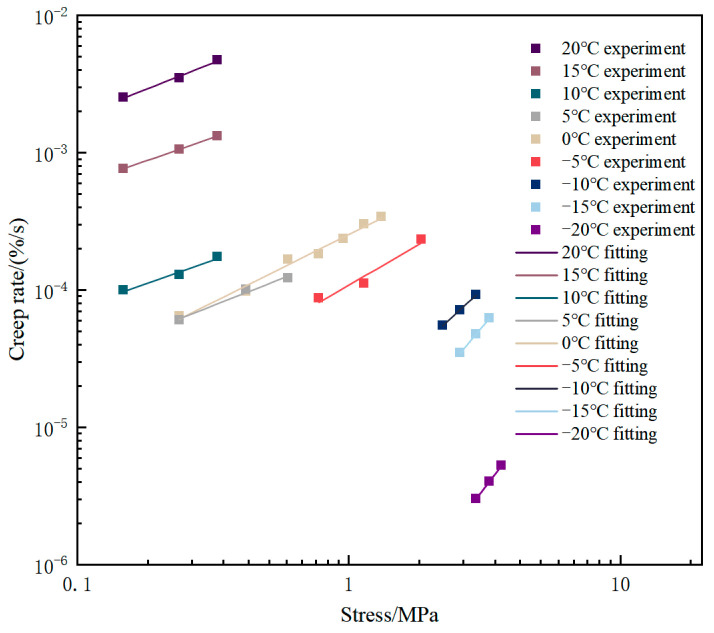
Creep rate–temperature–stress plot in double logarithmic coordinates.

At low temperature, a higher creep rate indicates stronger deformation ability and greater toughness, and then better resistance to low-temperature cracking. The lower the creep rate under high-temperature conditions, the stronger the ability of asphalt concrete to resist high-temperature deformation [27]. To quantify the creep rate of asphalt concrete, the steady bending creep rate of asphalt concrete under different temperatures and stresses can be obtained by linear fitting of the linear phase of the bending creep curve. Figure 6 shows the stress dependence of the steady-state creep rate of asphalt concrete at various temperatures.

At constant temperature, steady-state creep rate positively correlates with loading stress. In the case of low temperature and high stress, the slope of the fitted line is significantly larger, indicating that the stress has a significant effect on the creep rate. Taking −15 °C as an example, when the stress increases from 2.5776 MPa to 3.2976 MPa, the creep rate increases from 2.997 × 10^−6^%/s to 5.245 × 10^−6^%/s, and the creep rate increases 1.8 times with the stress. However, under low-stress conditions, the effect of stress growth on the creep rate is not obvious, and the creep rate increases with the increase in stress in approximately equal proportion. Taking 0 °C as an example, when the stress increases from 0.2376 MPa to 1.3176 MPa, the creep rate increases from 6.419 × 10^−5^%/s to 3.392 × 10^−4^%/s, and the creep rate increases six times with the stress. The above phenomena indicate that stress has an effect on the creep rate, and this effect is more significant at high stress levels. The creep rate is also affected by temperature, and shows a significant increase with the increase in temperature. It can be seen from the constant-stress-level creep tests conducted at 10 °C, 15 °C, and 20 °C that the creep rate increases significantly with the increase in temperature and even changes by orders of magnitude. Under the condition of the same stress of 0.2376 MPa, compared with the creep rate at 10 °C and 20 °C, the creep rate increases by 166 times when the temperature increases by 10 °C, which fully indicates that the temperature has a significant effect on the creep rate.

As can be seen from Table 6, coefficients *A* and *n* vary significantly with temperature. The coefficient *A* increases with increasing temperature. Under the temperature change of −20 °C to 20 °C, log*A* increases from −6.7184 to −1.95612 when the temperature increases by 40 °C, and the coefficient *A* changes by 6 orders of magnitude. On the contrary, the coefficient *n* decreases with the increase in temperature. Its variation ranges from 0.77958 to 2.55179, which decreases by about 2.5 times.

**Figure 7:**
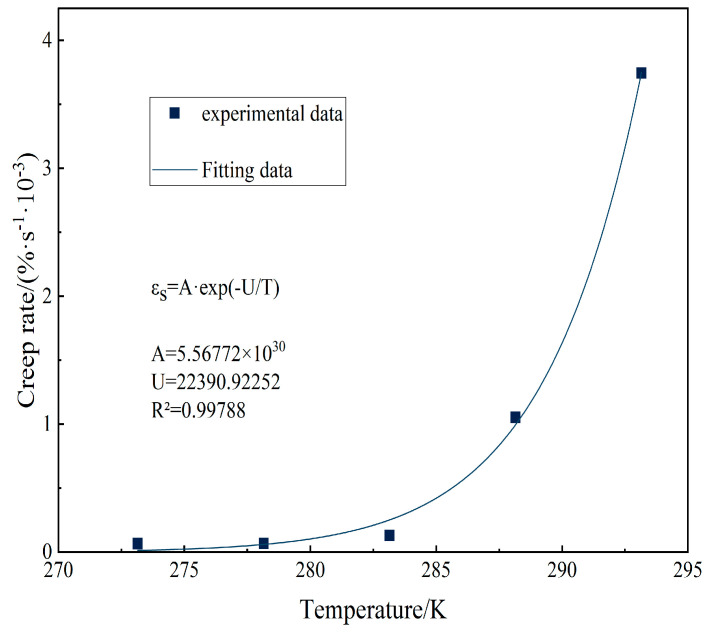
Creep rate is temperature-dependent.

According to thermal fluctuation theory, the creep rate of an object at absolute temperature *T* is precisely described by the Arrhenius formula, which is defined below. This formula establishes the quantitative relationship between the creep rate and temperature and provides a tool for understanding and predicting the creep rate of materials. Figure 7 shows the relationship between the creep rate and temperature obtained by fitting the Arrhenius formula. It can be seen from the figure that the test data are in good agreement with the fitting data, which further verifies the effectiveness and accuracy of the thermal fluctuation theory in the creep rate analysis [28].

(12) $$v={\;v}_{0}\exp(-\frac{\Delta U}{RT})$$

In the formula, the following are defined:

*v*—creep rate;

*v*_0_—material constant;

∆*U*—activation energy, J/mol;

R—universal gas constant, J/(mol·K);

*T*—absolute temperature.

**Figure 8:**
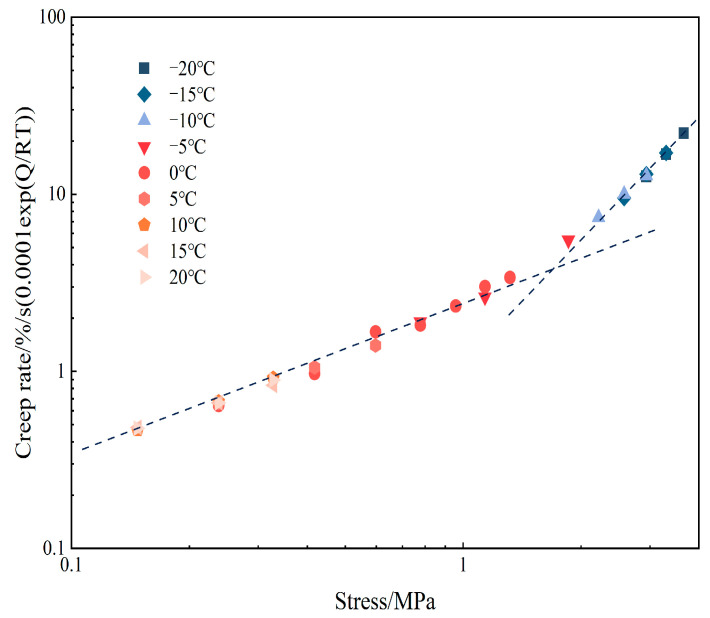
Normalized creep rate at 0 °C.

After clarifying the influence of temperature on creep rate, the test data at all temperatures were normalized with 0 °C as the reference temperature according to the Arrhenius formula. As can be seen from Figure 8, the relationship between steady-state creep rate and stress at the same temperature is not strictly linear in log–log coordinates but presents a turning point when the stress is about 2.5 MPa. This phenomenon further confirms some previous studies that find that Formula (6) is valid only within a specific stress interval. Therefore, the coefficient n needs to be expressed in segments, and creep tests are performed separately under low- and high-stress conditions. For the creep rate under high-stress conditions, an exponential function is usually used to modify the creep rate to obtain a complete creep rate expression. The form of the hyperbolic sine function of the exponential modified is more uniform, takes into account a variety of creep mechanisms, and is more applicable, which is also the constitutive model chosen in this paper [29].

### 4.2. Prediction of Long-Term Creep Behavior Based on Temperature-Dependent Constitutive Model

#### 4.2.1. Temperature-Dependent Constitutive Model

**Figure 9:**
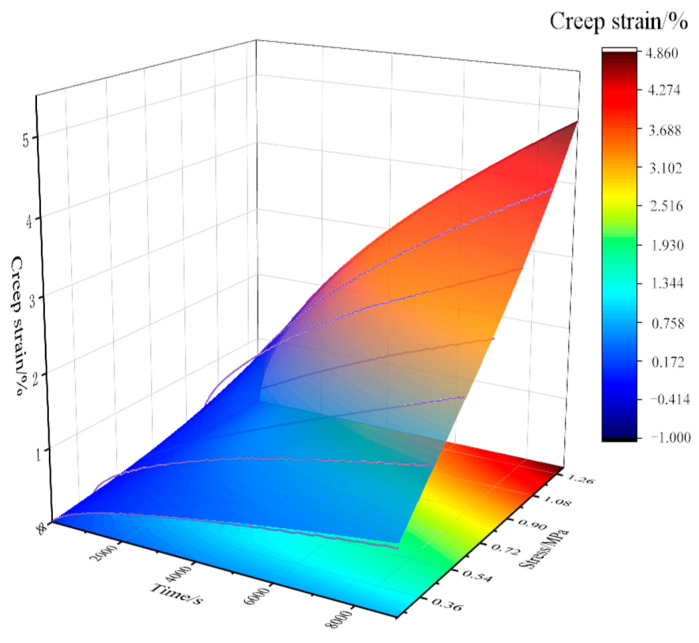
0 °C Findley model fitting results.

Based on Formula (8), the nonlinear surface fitting method is adopted to fit all creep curves of asphalt concrete at 0 °C [30]. At the same time, the stress–strain data at −15 °C are used to correct the nonlinear relationship, and *a*, *m*, and *n* are determined to be 1.30 × 10^−2^, 0.83079, and 0.58981, respectively. And the fitting surface as shown in Figure 9 is drawn. Figure 9 shows that the Findley model’s fitting results to the creep curve of asphalt concrete are very ideal; most of the scattered points fall on the fitting surface, and the coefficient of determination R^2^ is as high as 0.99.

**Figure 10:**
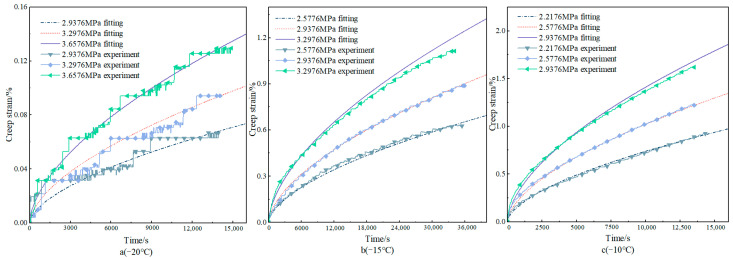

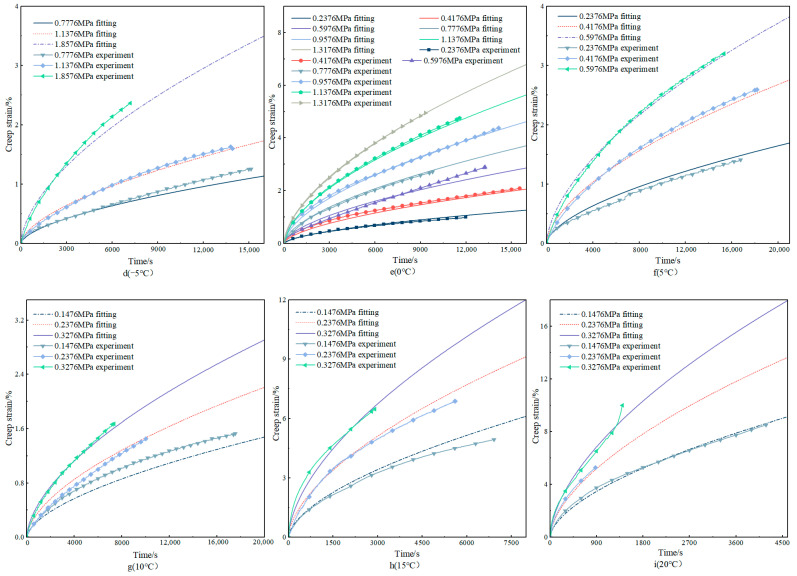
Findley model fitting results.

Surface fitting of creep curves at other temperatures based on the parameters *a*, *m*, and *n* were determined at 0 °C, and Figure 10 shows the model results compared to the test results. From the fitting results, it can be seen that the Findley model shows a good fit at several temperatures, and the coefficients of determination R^2^ are all 0.95.

**Figure 11:**
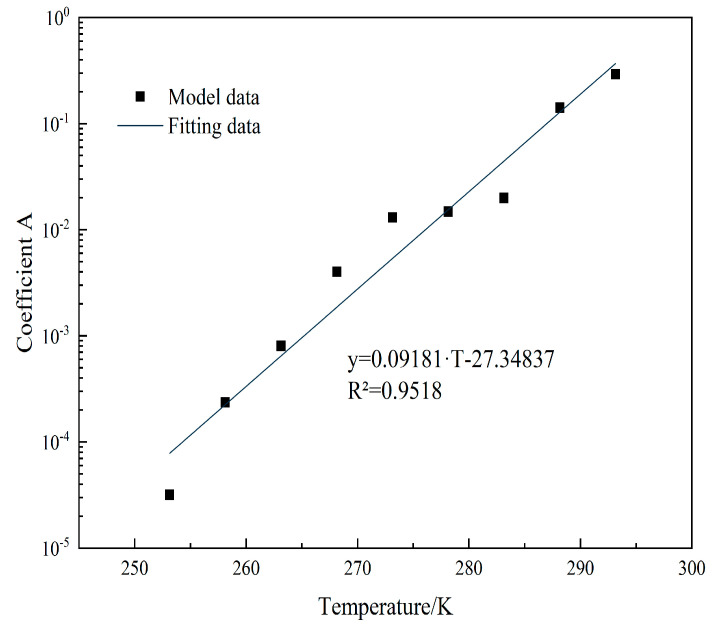
Findley model parameter A correlates with temperature.

To determine the temperature effect function, the coefficient *A* was fitted using the Arrhenius formula, and the fitting results are shown in Figure 11. Ultimately, a time–temperature–stress-dependent creep constitutive model was obtained as the general Findley model [31].

**Figure 12:**
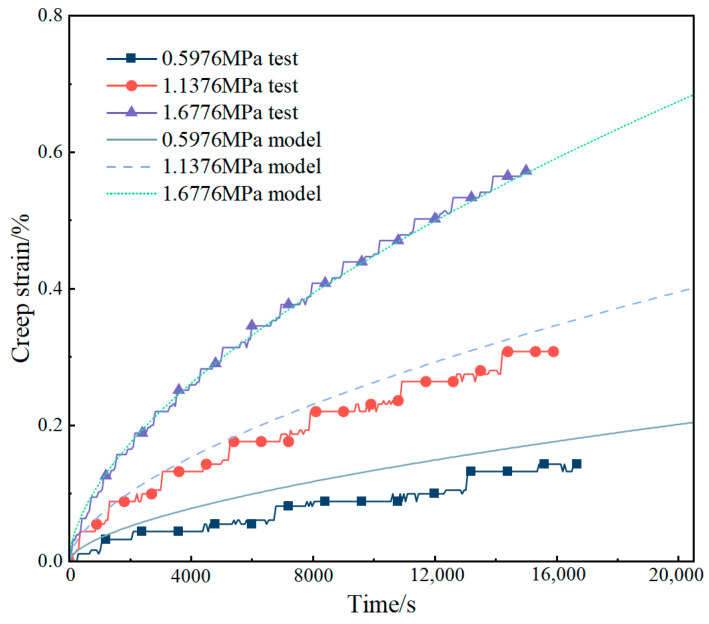
Comparison of the creep test results of asphalt concrete with the model at −10 °C.

To verify the correctness of the general Findley model, three additional independent tests were conducted at −10 °C. The test conditions are as follows: test 1 (σ = 0.5976 MPa), test 2 (1.1376 MPa), and test 3 (σ = 1.6776 MPa). These test data have not been used to fit the constitutive parameters. Thus, they constitute an independent validation dataset for assessing the accuracy of model predictions. Figure 12 shows the comparison between the test curve of the three creep tests and the model prediction curve. It can be seen from the figure that the model data agree well with the test data in a large stress range. The curve of test 1 is consistent with the predicted curve of the model. Although the results of test 2 and test 3 are slightly lower than the predicted curve of the model, the overall trend is consistent. Considering that asphalt concrete itself is a non-homogeneous composite material, especially at low temperatures with slow creep behavior and difficult test data collection, the impact of these systematic errors on the prediction accuracy is acceptable [32]. In summary, the general Findley model makes relatively accurate predictions and is suitable for a wide range of stress and temperature conditions.

#### 4.2.2. Prediction of Long-Term Creep Behavior

**Figure 13:**
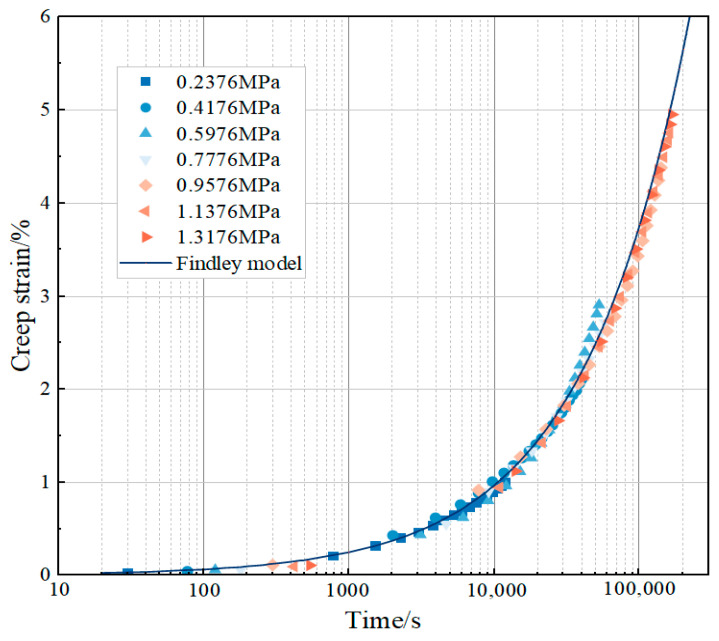
Creep curves and main curves at 0 °C and 0.2376 MPa.

To predict the long-term creep behavior of asphalt concrete at low stress levels at 0 °C, based on the time–stress equivalence principle, a creep master curve was constructed by equivalent curve offset using stress-accelerated short-term creep tests [33]. As shown in Figure 13, a reference stress of 0.2376 MPa was selected, and the creep curves of other stress levels were moved along the logarithmic time axis. Ultimately, a high-quality superposition of creep curves was achieved. It is proved that the creep curve is not affected by the stress level, and the time–stress equivalence principle applies to the construction of the master curve using short-term creep data. Based on the creep master curve results, a creep strain of 6% was predicted after 64 h of loading. Based on the Findley model, the predicted creep strain of asphalt concrete was 6% after 62 h at 0.2376 MPa stress. The predicted results of the time–stress superimposed master curve were essentially the same as the Findley model result.

**Figure 14:**
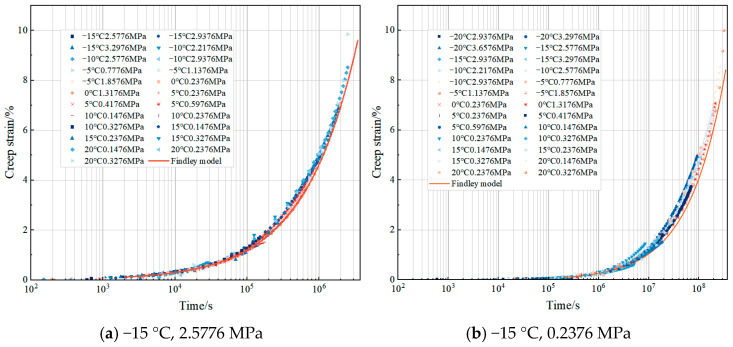
Creep curve and main curve at −15 °C.

The long-term deformation of asphalt concrete at low temperatures is predicted based on the results of creep tests at different stress and temperature levels. Figure 14a shows the creep master curve at the reference conditions (T = −15 °C, σ = 2.5776 MPa), by which it is predicted that the creep strain will reach 7% after 555 h of operation at the reference conditions. The Findley model predicts that the creep strain will reach 7% after 560 h. The results of the two forecasting methods are highly consistent. Given the limitations of experiment data at low temperatures, the creep curve of asphalt concrete at −15 °C and 0.2376 MPa was obtained by extrapolating the Findley model. The creep behavior of asphalt concrete at 0.2376 MPa is predicted based on Findley’s model in conjunction with the TTSSP, and the predicted results are shown in Figure 14b. The results show that the creep strain is expected to increase to 8% after 8 years. This prediction result not only validates the effectiveness of the TTSSP in the construction of creep main curves but also provides a reference for the long-term performance evaluation of asphalt concrete in practical engineering. However, predictions using the Findley model alone show that the creep strain is estimated to be 8% after 10 years. In long-term creep prediction, there are some deviations between these two methods. The reason may be that the Findley model fails to consider the acceleration stage of creep deformation when describing creep behavior, and the accelerated creep deformation in the later stage develops rapidly, resulting in deviations in long-term prediction.

## 5. Conclusions

This paper investigated the creep behavior of asphalt concrete at −20–20 °C using the bending creep test to study the influence of temperature and stress on creep behavior. The constitutive model considering the temperature effect is fitted to predict the long-term creep behavior at low temperatures and low stress combined with the time–temperature equivalent principle. The main conclusions are as follows:

1. The creep behavior of asphalt concrete exhibits significant temperature and stress dependence. With the increase in loading stress or the increase in temperature, the creep strain of asphalt concrete at the same time increases, while the creep rate also shows an accelerating trend, especially in high-stress conditions, showing obvious nonlinear creep behavior.
2. Elevated stress levels or temperature increases accelerate the creep process of asphalt concrete. The experimental results demonstrate that under the condition of 0.2376 MPa, the temperature increased from 0 °C to 20 °C, and the strain at the creep time of 9330 s nearly increased by 24 times. The loading stress increased from 0.2376 MPa to 1.3176 MPa at 0 °C, and the strain nearly increased by six times at the creep time of 880 s.
3. This study verified the applicability of the Findley constitutive model in investigating the mechanical properties of asphalt concrete facings. Based on this model, creep curve extrapolation was performed to simulate the creep deformation behavior of asphalt concrete under variable temperature–stress regimes. The findings provide scientific and systematic theoretical support for the long-term performance evaluation and durability design of asphalt concrete structures in complex environments, and is able to effectively simulate and predict the creep deformation of asphalt concrete in actual projects.
4. The long-term creep behavior of asphalt concrete under low-temperature conditions was predicted by combining the Findley constitutive model and TTSSP. Under the stress conditions of −15 °C and 0.2376 MPa, it takes nearly 8 years for asphalt concrete to reach 8% of the creep strain. This phenomenon clarifies the creep deformation law of asphalt concrete under long-term low-temperature loads: the low-temperature environment significantly delays the creep deformation rate of asphalt concrete, but the long-term strain accumulation still poses a potential risk to the structural stability. This prediction result can provide a theoretical basis for the structural design optimization of asphalt concrete facings in actual engineering, ensuring that they maintain sufficient bearing capacity and stability within the expected service life.

## Figures and Tables

**Table 1:** Quality evaluation result of coarse aggregate.

Index	Unit	Technical Requirements	Experimental Results
Apparent Density	g/cm^3^	≥2.6	2.710
Grade of Adhesion to Asphalt	Grade	≥4	5
Water Absorption	%	≤2	0.48
Durability	%	≤12	1.3
Heat Resistance	--	No cracking, decomposition	Qualified

**Table 2:** Quality identification results of fine aggregates.

Index	Unit	Technical Requirements	Limestone	Description
Apparent Density	g/cm^3^	≥2.55	2.734	--
Grade of Adhesion to Asphalt	%	≤2	0.4	--
Water Stability Grade	grade	≥6	9	Sodium carbonate solution boiled for 1 min
Organic Matter and Soil Content	%	≤2	0.0	--

**Table 3:** Filler quality identification results.

Index	Unit	Technical Requirements	Experimental Results
Apparent Density	g/cm^3^	≥2.5	2.717
Hydrophilicity Coefficient	--	≤1.0	0.6
Water Absorption	%	≤0.5	0.2
Fineness < 0.6 mm	%	100	100
Fineness < 0.15 mm	%	>90	100
Fineness < 0.075 mm	%	>85	100

**Table 4:** Asphalt quality appraisal results.

Index	Unit	Quality Indicator	Detection Result
Penetration (25 °C, 100 g, 5 s)	1/10 mm	60~80	68.1
Ductility (5 cm/min, 15 °C)	cm	≥150	>150
Ductility (1 cm/min, 4 °C)	cm	≥15	41.3
Softening Point	°C	48~55	48.1
Density (25 °C)	g/cm^3^	Measured	0.986
The Mass Change of Film After Oven Experiment	%	±0.8	−0.10
Penetration Ratio (25 °C) After Film Oven Experiment	%	≥61	80.6
Film Oven Experiment Ductility (5 cm/min, 15 °C)	cm	≥80	>100
Film Oven Experiment Ductility (1 cm/min, 4 °C)	cm	≥4	16.7

**Table 5:** Creep test parameters.

Experimental Conditions	Experimental Temperature, °C	Absolute Temperature, K	Loading Stress, MPa
1	−20	253.15	2.9376, 3.2976, 3.6576
2	−15	258.15	2.5776, 2.9376, 3.2976
3	−10	263.15	2.2176, 2.5776, 2.9376
4	−5	268.15	0.7776, 1.1376, 1.8576
5	0	273.15	0.2376, 0.4176, 0.5976, 0.7776, 0.9576, 1.1376, 1.3176
6	5	278.15	0.2376, 0.4176, 0.5976
7	10	283.15	0.1476, 0.2376, 0.3276
8	15	288.15	0.1476, 0.2376, 0.3276
9	20	293.15	0.1476, 0.2376, 0.3276

**Table 6:** Creep rate linear fitting results.

Temperature/°C	log*A*	*A*	*n*	R^2^
20	−1.95612	0.011063	0.77958	0.99309
15	−2.54703	0.002838	0.68655	0.99982
10	−3.43575	0.000367	0.69494	0.97522
5	−3.59647	0.000253	0.98729	0.9863
0	−3.72618	0.000188	0.77512	0.98323
−5	−3.96487	0.000108	1.14454	0.95634
−10	−5.05639	8.78 × 10^−6^	1.96361	0.99814
−15	−4.95713	1.1 × 10^−5^	0.84855	0.99995
−20	−6.7184	1.91 × 10^−7^	2.55179	0.99983

## Data Availability

The data used to support the findings of this study are available from the corresponding author upon request.
